# EEG oscillations during sleep and dream recall: state- or trait-like individual differences?

**DOI:** 10.3389/fpsyg.2015.00605

**Published:** 2015-05-07

**Authors:** Serena Scarpelli, Aurora D’Atri, Maurizio Gorgoni, Michele Ferrara, Luigi De Gennaro

**Affiliations:** ^1^Department of Psychology, Sapienza University of Rome, Rome, Italy; ^2^Department of Life, Health and Environmental Sciences, University of L’Aquila, L’Aquila, Italy

**Keywords:** dream, frontal cortex, theta activity, EEG topography, episodic memory, continuity hypothesis

## Abstract

Dreaming represents a peculiar form of cognitive activity during sleep. On the basis of the well-known relationship between sleep and memory, there has been a growing interest in the predictive role of human brain activity during sleep on dream recall. Neuroimaging studies indicate that rapid eye movement (REM) sleep is characterized by limbic activation and prefrontal cortex deactivation. This pattern could explain the presence of emotional contents in dream reports. Furthermore, the morphoanatomical measures of amygdala and hippocampus predict some features of dream contents (bizarreness, vividness, and emotional load). More relevant for a general view of dreaming mechanisms, empirical data from neuropsychological and electroencephalographic (EEG) studies support the hypothesis that there is a sort of continuity between the neurophysiological mechanisms of encoding and retrieval of episodic memories across sleep and wakefulness. A notable overlap between the electrophysiological mechanisms underlying emotional memory formation and some peculiar EEG features of REM sleep has been suggested. In particular, theta (5–8 Hz) EEG oscillations on frontal regions in the pre-awakening sleep are predictive of dream recall, which parallels the predictive relation during wakefulness between theta activity and successful retrieval of episodic memory. Although some observations support an interpretation more in terms of an intraindividual than interindividual mechanism, the existing empirical evidence still precludes from definitely disentangling if this relation is explained by state- or trait-like differences.

## Introduction

Over the centuries, dreaming has been studied from multiple points of view, but only recently it has become a neuroscientific object of study. A crucial step for the development of the scientific study of dreams is represented by the discovery of rapid eye movement (REM) sleep stage (Aserinsky and Kleitman, 1953; Dement and Kleitman, 1957a,b). Early results showed that in 80% of the awakenings from REM sleep the subjects reported at least one dream (Dement and Kleitman, 1957a,b). This finding gave rise to the well-known REM = dreaming equation, which dictated empirical dream research for years. The assumption that dreaming is just an epiphenomenon of REM sleep was refuted by Foulkes (1962). He showed that people report dream mentation also when awakened from non-REM (NREM) sleep. Furthermore, subjects were awakened just before the first REM period, pointing to a dream recall also during a sleep period without REM (Foulkes, 1962).

Neuropsychological evidence confirmed that REM sleep and dream recall are modulated by two different brain mechanisms. Patients affected by brainstem lesions in the regions related to REM sleep generation still preserved their dream recall (Solms, 1997, 2000). Conversely, dream recall totally disappeared in patients with focal forebrain lesions and no impairment of REM sleep (Solms, 2000). This double dissociation between REM and dreaming allows us to state that dream recall is possible both after REM and NREM sleep.

Although the dichotomy REM = dreaming/NREM = non-dreaming is outdated, the differentiation between the two types of dream reports generated a “new dichotomy” between REM and NREM dream contents, that still influences the dream research. Mental activity from REM sleep is commonly defined “dream-like,” and it is characterized by emotional load, bizarreness, and vividness (Foulkes and Rechtschaffen, 1964; Foulkes, 1967; Antrobus, 1983; Foulkes and Schmidt, 1983; Waterman et al., 1993; Stickgold et al., 1994; Casagrande et al., 1996). Differently, dream report from NREM sleep is typically “thought-like” (Foulkes, 1967); in this case, the mental activity is less emotionally intense and the contents are closer to reality and more fragmented than dream-like reports (Rechtschaffen et al., 1963; Foulkes, 1967). It should be noted that also this “new dichotomy” has been questioned. Indeed, a significant portion of dreams reported from NREM stages shows several features peculiar of “dream-like” mentation (Monroe et al., 1965; Zimmerman, 1970; Solms, 2000). Furthermore, it has been observed that the control for word count makes relatively similar REM and NREM reports (Antrobus, 1983; Foulkes and Schmidt, 1983; Cavallero et al., 1992).

As a result, dream research was focused on the physiological state underlying dreaming, both in REM and NREM sleep. Many studies have been performed by awakenings from specific sleep stages, to explore the differences among the neurobiological (electroencephalographic, EEG) correlates of dreaming. This line of research has stimulated three different models in a bid to explain the mechanisms that generate dreaming. The first model assumed that there is “one-generator” to produce sleep mentation in all sleep stages (Foulkes, 1985). This model posits that the different qualitative features between REM and NREM sleep mentation depend on memory activation, organization of mnestic material in a congruent narrative structure and conscious interpretation. The cortical synchronization during NREM sleep (less cerebral activation) does not allow a complex processing and consolidation of dream contents (Nielsen, 2000). This view is consistent with some studies showing that the differences between REM and NREM dreams only concern quantitative features (Antrobus, 1983; Foulkes and Schmidt, 1983; Cavallero et al., 1992). Furthermore, some studies reported that “dream-like” sleep mentation is detected also closer to sleep onset and to awakening, in correspondence of higher cortical activity (Foulkes, 1962; Foulkes and Vogel, 1965).

Conversely, Hobson and McCarley (1977) supported a “two-generator” model for REM and NREM sleep mentation. According to this perspective, dream contents should be reflected by the peculiar physiological features of the sleep *scenario*. Hence, this could explain the different features of REM and NREM dream reports (Hobson et al., 2000). In an attempt to integrate these two models, Nielsen (2000) proposed a “covert-REM” model. He defined “covert-REM” any NREM sleep episode including some distinctive REM sleep processes (i.e., muscle atonia, rapid eyes movements), which cannot be scored as REM sleep according to the standard criteria. For instance, these episodes may occur during wake-sleep transitions (i.e., sleep onset) or when REM sleep episode skips the first sleep cycle; moreover, “covert-REM” could appear in NREM sleep in proximity (within 15 min) of the subsequent REM sleep episode (Nielsen, 2000). This view could be considered as an extension of the two-generator model, because it supports the hypothesis that dreaming is possible just during a particular NREM sleep stage that embodies some characteristics peculiar of REM sleep (Nielsen, 2000).

It should be emphasized that the empirical studies in support of these models are characterized by a basic limitation, that is the assumption that the dream content is generated in the same stage upon which subject has been awakened. The issue of dream *generation* has affected the physiological approach to dream study for a long time. For instance, neuroimaging investigations (Nofzinger et al., 1997; Maquet et al., 2005) are based on this methodological bias, still considering dreaming intrinsically related to REM sleep and not providing a systematic evaluation of the relations between sleep mentation and cerebral activation patterns before awakenings from different sleep stages (De Gennaro et al., 2012).

More recently, some empirical findings have revitalized the neurobiological approach to dream recall by investigating the association between the electrophysiological pattern in the segment of sleep closer to final awakenings and presence/absence of dream reports, rather than focusing on an hypothetical time of dream generation (Corsi-Cabrera et al., 2003; Takeuchi et al., 2003; Esposito et al., 2004; Chellappa et al., 2009, 2011; Marzano et al., 2011). Quantitative EEG analysis aims to establish correlations between some dream-cognitive features and the brain states prior to awakening. Within some contrasting results, the most consistent finding points to a continuity between the electrophysiological brain mechanisms involved in the encoding of episodic memory across wakefulness and sleep state (Takeuchi et al., 2003; Esposito et al., 2004; Chellappa et al., 2011; Marzano et al., 2011). In particular, the EEG oscillations predictive of subsequent successful memory performance in wakefulness resemble the EEG frequencies and topography detected before successful dream recall, both from REM and NREM sleep (Takeuchi et al., 2003; Esposito et al., 2004; Chellappa et al., 2011; Marzano et al., 2011).

This review focuses on the EEG patterns related to dream recall, with specific reference to recent empirical evidence about the relationship between theta oscillations and episodic memory. The ultimate purpose is to discuss the issue of whether the predictive relationship between EEG oscillations and dream recall can be interpreted in terms of “state- or trait-like” differences. Namely, the neurobiological correlates of a successful recall may depend on the physiological *scenario* related to the specific sleep stage of awakening (“state-like” factors), or they could be linked to interindividual differences that steadily characterize recallers and non-recallers (“trait-like” factors).

## Theta Brain Oscillations Involved in Episodic Memory During Wakefulness

Several studies have reported that memory processing is modulated by specific brain oscillations (Klimesch et al., 1996; Başar et al., 1999; Klimesch, 1999). Recent evidence indicates that EEG activity could predict the subsequent cognitive performance (Paller and Wagner, 2002; Nyhus and Curran, 2010; Addante et al., 2011). In particular, theta rhythm, traditionally defined in the 4–8 Hz range (Niedermeyer and Lopes da Silva, 1993), seems to correlate consistently with episodic memory (Klimesch et al., 1996; Klimesch, 1999), described as the ability to remember past experiences and autobiographical events (Tulving, 2002).

Theta rhythm may be considered as the local field potential (LFP) that represents an electrophysiological marker of hippocampal activity (Rutishauser et al., 2010), both during awake behavior and REM sleep (Klimesch, 1996; Cantero et al., 2003). Theta activity modulates neuronal changes in hippocampal formation and in neocortical structures (Mitchell et al., 2008). In this regard, many researchers have tried to explain the relationship between hippocampal and cortical theta, although the exact mechanisms involved in this interaction are still uncertain (Klimesch, 1996; Lega et al., 2012). Moreover, field theta oscillations are not always coherent with hippocampal theta (Kahana et al., 1999; Raghavachari et al., 2001). Some authors have suggested that cortical theta may be induced via the extensive projections from the hippocampus to the neocortex, described as hippocampo-cortical feedback loops (Miller, 1991; Klimesch, 1996).

In humans, studies on hippocampal theta have been performed by intracranial electrodes (intracranial-EEG, iEEG) in neurosurgical patients while engaged in cognitive tasks, or were focused on cortical theta by scalp recordings [EEG or magnetoencephalography (MEG); Nyhus and Curran, 2010]. Scalp EEG recordings in humans have shown that theta power increases during memory tasks (Hsieh and Ranganath, 2014). Specifically, the role of theta activity both in encoding and retrieval of episodic information has recently received considerable attention (Klimesch et al., 1997, 2001; Klimesch, 1999; Molle et al., 2002; Guderian and Düzel, 2005; White et al., 2013). Klimesch et al. (1996), using an event related synchronization/desynchronization (ERS/ERD) approach, investigated differences in EEG power between the encoding phase of information that should be correctly remembered and not remembered later. During the encoding, subjects did not know that they would be subsequently retested. The authors found that theta activity increases especially during encoding of items subsequently recalled. Further results from the same group confirmed that changes in theta power were indicative of good or bad performance in retrieval memory (Klimesch et al., 1997; Doppelmayr et al., 1998; Klimesch, 1999). Later studies have demonstrated that theta activity increases also during retrieval of previously studied information (Klimesch et al., 2000, 2006). These works have emphasized that theta enhancement is larger during recognition than encoding phase.

Similarly, Molle et al. (2002) found that an efficient recognition of words and faces was correlated with theta synchronization (and alpha desynchronization) as compared to bad performance.

More interestingly, some studies have observed that also theta oscillations prior to the effective stimulus presentation are related to an efficient episodic encoding or retrieval (Guderian et al., 2009; Addante et al., 2011; Gruber et al., 2013). Guderian et al. (2009) reported that high pre-stimulus theta oscillations were related to a successful recall. Similarly, Addante et al. (2011) found a frontal theta enhancement during pre-stimulus phase associated to a subsequent successful retrieval. In other words, these results support the view that memory performance depends on state-related physiological factors *before* the beginning of the task (Addante et al., 2011).

Evidences from MEG recordings are coherent with these findings. Results point to differential patterns between recall and non-recall conditions associated to memory tasks (Jensen and Tesche, 2002; Osipova et al., 2006). In particular it was found that encoding and recall of picture stimuli are linked to theta oscillations in the right hemisphere (Osipova et al., 2006).

Intracranial EEG studies have confirmed that theta oscillations have a role in memory formation (Sederberg et al., 2003; Rutishauser et al., 2010; Lega et al., 2012). An increase of theta activity in temporal and right frontal lobes correlated with successful recall of lists of words (Sederberg et al., 2003). Hippocampal/parahippocampal-cortical theta phase-coupling has also been investigated by iEEG recording in neurosurgical patients (Fell et al., 2003; Anderson et al., 2010; Rutishauser et al., 2010; Lega et al., 2012). Rutishauser et al. (2010) showed that an optimal memory performance was predicted by a tight coordination of spike timing in correspondence of the local theta oscillations. In addition, higher theta hippocampal-cortical phase-coupling was related to successful retrieval in a memory task (Lega et al., 2012).

The above-mentioned increase of theta activity has been typically reported over the frontal areas (Klimesch et al., 1996, 1997, 2006; Jensen and Tesche, 2002; Molle et al., 2002). Specifically, this phenomenon occurs over Fz, F3, F4 channels, and it is known as “frontal midline theta” (FMT; Ishihara and Yoshi, 1972; Klimesch et al., 2006; Mitchell et al., 2008; Hsieh and Ranganath, 2014).

It is well known that the prefrontal cortex (PFC) is involved in memory processes (Grady et al., 2003; Gazzaley et al., 2004; Nee and Jonides, 2008). In line with this finding, an innovative neuroimaging study, by using diffusion tensor imaging (DTI), demonstrated that the relationship between theta and memory depends on hippocampus-prefrontal cortex connectivity by showing that participants with a remarkable connectivity had better long-term memory (Cohen, 2011). These subjects exhibited EEG patterns characterized by theta and delta oscillations over the frontal regions. In addition, iEEG recordings also showed that theta activity modulates the interaction between PFC and the medial temporal lobe (MTL), suggesting that both these areas may be part of the same network engaged in recall operations (Anderson et al., 2010). In particular, MEG studies have shown that the frontal theta rhythm could be generated by the medial prefrontal cortex (mPFC) and the anterior cingulate cortex (ACC; Asada et al., 1999; Ishii et al., 1999; Nishida et al., 2004). These regions seem to play a central role in selective and continuous attention during working memory and recall tasks (Cohen et al., 1997; Petit et al., 1998; Smith and Jonides, 1999; Rowe et al., 2000).

However, it should be underlined that some studies found increased theta coherence between intracranial electrodes during the encoding of subsequently recognized items, without observing any variation in theta power (Fell et al., 2003). In some cases, the frontal theta correlated negatively with retrieval and positively with forgetting (Hanslmayr et al., 2010; Staudigl et al., 2010; Khader and Rösler, 2011; Lega et al., 2012; Pastötter and Bäuml, 2014). For instance, Staudigl et al. (2010) reported an increased FMT related to the increased interference during episodic retrieval task. Recent works suggested that it might be useful to consider two sub-ranges of theta activity, i.e., slow theta (∼3 Hz) and fast theta (∼7 Hz) oscillations (Lega et al., 2012; Pastötter and Bäuml, 2014). These bands differently affected memory performance since slow cortical and hippocampal theta oscillations were positively related to an optimal retrieval, while fast cortical and hippocampal theta oscillations were negatively related to it (Lega et al., 2012; Pastötter and Bäuml, 2014).

Frontal theta oscillations have been observed also during meditation and state of drowsiness (Takahashi et al., 1997; Kubota et al., 2001). In this respect, Norman et al. (2007) proposed an “Oscillating Inhibition Model,” which posits that theta rhythm may represent a mechanism for inhibition of useless items in memory system. In other words, theta activity could help to suppress the external interference with selective retrieval of target memory during cognitive tasks or in meditative state, while subjects are focused on inner experiences.

Finally, it should be considered that EEG power in the theta band is strictly associated to subjective sleepiness and sleep propensity (Finelli et al., 2000; Tinguely et al., 2006; De Gennaro et al., 2007; Marzano et al., 2007; Gorgoni et al., 2015). Indeed, an increase of theta power during extended wakefulness has been demonstrated (Cajochen et al., 1996; Aeschbach et al., 1997; Finelli et al., 2000). In this respect, the cognitive performance, especially memory function, may be affected by the time of data collection. Therefore, it is necessary underline that circadian and homeostatic modulation on theta activity should be taken into account -at least, by maintaining them constant across conditions- as possible confounding variables.

Summing up, the most studies seem to confirm that theta oscillations could be predictive of memory performance. It should be noted that a relatively high theta activity is commonly detected in REM sleep (Marzano et al., 2010). In particular, the theta band shows a fronto-central gradient with highest values over the midline regions (Marzano et al., 2010). Furthermore, several studies suggest a link between REM sleep and memory consolidation (Siegel, 2001; Nishida et al., 2009). For instance, a relationship between prefrontal theta rhythm during REM sleep and emotional memory consolidation has been reported (Nishida et al., 2009).

Keeping in mind these considerations, we hypothesize that the analysis of the changes in spectral power during sleep, in association with the specific mental activity of sleep (i.e., dreaming), might give a significant contribution in understanding the role of theta band in cognitive processes.

## Neurobiological Approach to Dream Recall

In the last decades, there has been a growing interest in the neurobiological correlates of dreaming. Considerable strides in dream research have been made mostly thanks to the investigations in brain damage patients, as well as to the employment of brain imaging techniques and quantitative analysis of polysomnographic (PSG) measures.

However, it should be emphasized that dreaming is a peculiar object of study, especially because it is impossible to access directly to sleep mental contents. We can access dream content only by awakening the subject: therefore, the real object of study is the dream recall, rather than dreaming in itself. It follows that dream report is inevitably influenced by two states of consciousness, wakefulness and sleep. We may consider dream recall as a particular type of mental activity, which shares some features with encoding and retrieval of episodic memories during daytime activity (Marzano et al., 2011).

### Neuropsychological Findings

The first neuropsychological studies on dreaming aimed at evaluating the correlations between dream features in brain damaged patients and anatomical lesion sites.

Some studies have explored the different role played by the brain hemispheres in sleep mentation. Originally, it was hypothesized that dreaming depends on the right hemisphere. However, a pionieristic meta-analysis refuted this view (Doricchi and Violani, 1992). It was highlighted that also subjects who have undergone a right hemispherectomy reported dream contents relatively similar to healthy people (Doricchi and Violani, 1992). Furthermore, it was evidenced that frontal lobe damage was not systematically associated to dream loss; instead, parietal lesions caused complete dream cessation. This phenomenon was observed both as a consequence of unilateral and bilateral lesions. With respect to the specific issue of laterality, the authors hypothesized that right hemisphere is linked to dream materials and left hemisphere to dream encoding and interpretation (Doricchi and Violani, 1992).

Further investigations have demonstrated that total dream loss is also observed in patients affected by ventromesial frontal white matter damages (Solms, 1997). It should be noted that this area corresponds to the region affected by prefrontal lobotomy. The 80% of lobotomized subjects did not report mental activity during sleep (Frank, 1946; Partridge, 1950; Solms, 1997). Consistently, empirical evidences have underlined that the chemical stimulation of this area by L-dopa elicits psychotic symptoms, nightmares and more vivid dream contents (Scharf et al., 1978; Nausieda et al., 1982).

Starting from brain damage studies, a nosology of dream activity alterations was implemented (Solms, 1997). Solms defined “*visual anoneria*” the condition characterized by mental sleep activity without visual components. This deficit was associated with temporo-occipital lesions (Brodmann 40 area). In this case, the patients showed visual impairment also in wakefulness, in particular on visuo-spatial tasks (Epstein and Simmons, 1983; Murri et al., 1985; Solms, 2000). The “*global anoneria*” consists of complete cessation of dreaming. Typically, this syndrome was associated with posterior cortical lesions, near the temporo-occipital-parietal junction or deep and bilateral frontal damage, in correspondence of the ventromesial region (Solms, 1997; Domhoff, 2011). Commonly, these patients show reiterations and disinhibition (Bischof and Bassetti, 2004). Again, these anomalies can be observed during spatial memory tasks in wakefulness. The third syndrome, “*anoneirognosis*,” concerns the impossibility to distinguish internal experiences (i.e., dream) from reality. This alteration may be observed in patients with limbic-frontal lesions, over mPFC, ACC, and basal forebrain. This syndrome is always associated to a general state of confusion during wakefulness. For instance, patients reported an intrusion of dream contents into waking thought (Solms, 1997; Domhoff, 2011). Furthermore, the “*recurring nightmares*” syndrome was typically found in correspondence of temporal-limbic seizures that sometimes are associated to paroxysms (Solms, 1997).

Brain damage studies are not free from limitations. Firstly, the mental sleep activity in these patients is never compared to dreaming during *premorbid* condition. This means that mental sleep activity before the brain damage have been investigated only by retrospective self-report (Schwartz and Maquet, 2002). Furthermore, it is quite common that patients take medications that may influence some cognitive mechanisms involved in dreaming (Gottesmann, 2002).

More recently, the use of neuroimaging techniques [i.e., positron emission tomography (PET) and functional magnetic resonance imaging (fMRI)] has allowed to substantially replicate the data obtained from anatomical studies. The early neuroimaging studies on dreaming were based on the assumption that dream contents reflect the activation level of neural structures that typically regulate cognitive processes. These studies, still bounded to the implicit assumption that sleep mentation strictly depends on REM sleep physiology, tried to find for each “dream-like” feature a relationship with specific activation/deactivation of brain areas.

Imaging studies mostly show that REM sleep is characterized by a general high cerebral activation (Maquet et al., 1996; Braun et al., 1997, 1998; Nofzinger et al., 1997; Maquet, 2000; Wehrle et al., 2007; Hong et al., 2009; Nir and Tononi, 2010). It was observed an increase of activity in the thalamus (Peigneux et al., 2001; Wehrle et al., 2005), hippocampal formation (Wehrle et al., 2007), visual cortex (Lövblad et al., 1999; Wehrle et al., 2007; Hong et al., 2009), orbitofrontal cortex (Maquet et al., 1996; Braun et al., 1997, 1998; Hong et al., 2009), amygdala and ACC (Maquet et al., 1996; Braun et al., 1997, 1998; Nofzinger et al., 1997).

It is well known that the amygdala and the hippocampal formation are involved in memory processes (Nofzinger et al., 1997; Braun et al., 1998). The activation of amygdala and hippocampus seems to play a putative role in the processing of emotional contents during REM sleep. In this regard, it should be noted that an important feature of dreaming is the alteration of mnestic processes: indeed, except for particular emotional contents, subjects have difficulties in retrieving their dream activity (Nir and Tononi, 2010). Some alterations in memory system are probably associated to the relative deactivation of the prefrontal cortex (Maquet et al., 1996, 2005). Typically, this area controls working memory, rational thought and executive functions (Nir and Tononi, 2010).

Furthermore, some results have demonstrated that the attenuation in the voluntary control on thoughts and actions during dreaming depends on the right inferior parietal cortex during REM sleep (Maquet et al., 1996; Braun et al., 1997). Such findings fit quite well with the “traditional” viewpoint that dream-like activity is strictly related to the REM sleep brain activation pattern. However, as previously mentioned, it should be underlined that these neuroimaging studies, with one notable exception (Maquet et al., 1996), have overlooked the actual presence of a successful dream recall, inferring relationships between neural structures and dream material.

### EEG Brain Oscillations Involved in Mental Activity During Sleep

Polysomnographic technique and laboratory awakenings are considered the *gold standard* method to investigate sleep mentation. After awakenings from NREM sleep, some studies have found a relationship between alpha activity and dream recall (Takeuchi et al., 2003; Esposito et al., 2004; Marzano et al., 2011), although the results are not always consistent. For instance, one study reported that dream recall correlated with a decrease of alpha activity in correspondence of central areas (Takeuchi et al., 2003). On the other hand, two more recent studies showed an increase of alpha oscillations in temporo-parietal regions (Esposito et al., 2004; Marzano et al., 2011). Differently, Chellappa et al. (2011) observed that dream report from NREM sleep is associated with a decline of delta and sigma EEG power over the centro-parietal regions.

Also dream recall after REM sleep is associated to alpha activity. In particular, a correlation between dream report and a decrease of alpha band on the frontal region has been reported (Esposito et al., 2004; Chellappa et al., 2011). However, these data are incoherent with the evidence that dream recall is predicted by an enhancement of theta activity in frontal area prior to awakening (Marzano et al., 2011). Keeping in mind the role played by the theta and alpha rhythms in the episodic memory formation and retrieval (Klimesch, 1996), it may be hypothesized a sort of continuity between the mechanisms that modulate episodic memory processes across sleep and wakefulness (Marzano et al., 2011). This is also in line with a psychological point of view which underlines that qualitative and quantitative aspects of sleep mentation are determined by waking-life experiences and feelings (Domhoff, 2003; Schredl, 2003, 2009).

To sum up, the neuroscientific literature on the EEG correlates of dream recall still depict an heterogeneous picture. We must bear in mind that the data have been obtained by partly different methodologies (such as time of awakening and procedures to collect dream content). Indeed, dream reports were collected after the first sleep cycle (Esposito et al., 2004), the entire night (Marzano et al., 2011), multiple nap (Chellappa et al., 2011), or REM sleep onset (SOREMP) and NREM sleep onset (NREMP) periods (Takeuchi et al., 2003). Consequently, they take in account partially different phenomena.

## State- or Trait-Like Differences?

It should be highlighted that the most of the empirical data on neural correlates of dreaming are based on *between-subjects* designs, making impossible to disentangle whether the patterns observed are state-like or trait-like. In order to reach a more complete knowledge about dreaming, it is necessary to clarify if dream recall is influenced by specific stable differences characterizing each subject (i.e., the trait-like hypothesis) or, alternatively if dream contents can only be retrieved in correspondence with specific brain activity strictly related to the physiological sleep *scenario* (i.e., the state-like hypothesis). Specifically, the first hypothesis implies that some individuals could show steady topographical EEG pattern, independently from dream recall or non-recall conditions. Hence, the trait-like relationship may be interpreted in terms of specific EEG features which allow subjects to stably recall or not recall dreams. On the other hand, the second hypothesis suggests that it is a recognizable EEG pattern during a specific sleep stage to determine or at least predict the subsequent dream recall at awakening.

Very few studies have tried to disentangle this issue. For instance, Marzano et al. (2011) evaluated if the EEG pattern observed in the last sleep segment prior to awakening was also evidenced during the previous nocturnal sleep cycles (i.e., if it was a stable pattern in entire night). They examined EEG power in correspondence of the midpoint of each sleep cycle (second, third and fourth episodes of REM and stage 2), and reported that significant differences in the EEG pattern between recallers (REC) and non-recallers (NREC) groups were detected only in proximity to the awakening time. Hence, those differences were not a stable pattern of EEG activity during the night (Marzano et al., 2011). However, the lack of systematic differences across all sleep episodes do not provide a definitive evidence on the state-or trait-like question.

A recent neuroimaging study investigated, by DTI technique, the association between qualitative and quantitative features of dream recall and morphoanatomical measures of the amygdala and hippocampus. In this approach, dream recall has been investigated according to a trait-like view, and correlated to interindividual differences of the subcortical structures. A correlation between the bizarreness of dreams and some microstructural features of the amygdala (smaller volume and lower structural integrity) was found (De Gennaro et al., 2011). Furthermore, emotional load was associated to a smaller volume of the left hippocampus and to a larger volume of the right hippocampus. Moreover, a relationship between emotional load and structural integrity of the left amygdala was reported (De Gennaro et al., 2011). These data support the hypothesis that the amygdala is related to the emotional encoding of dream contents, suggesting a continuity between the formation of emotional memory across wakefulness and sleep. The central role of the amygdala in the emotional regulation in wakefulness as in sleep is particularly clear in post-traumatic stress disorder (PTSD) patients. Chronic post-traumatic nightmares could be considered “the hallmark of PTSD” (Ross et al., 1989; Germain, 2013). Dream reports in PTSD patients are characterized by themes, vivid images and emotions associated to the traumatic events. It should be noted that many studies have found a hyperactivity of the amygdala in this disorder (Shin et al., 2006; Germain et al., 2008). Specifically, REM sleep could amplify the altered function of the amygdala in these patients, increasing dysphoric dreams (Germain et al., 2008).

“Trait-like” differences have also been considered in terms of “*dream recall frequency*” (DRF). A PET study demonstrated that High Recallers (higher DRF) show a greater regional cerebral blood flow (rCBF) in temporo-parietal junction (TPJ) during REM sleep, stage 3 and wakefulness than Low Recallers (lower DRF). It was also observed a higher rCBF in High Recallers in mPFC only during REM sleep and wakefulness (Eichenlaub et al., 2014a). These evidences are consistent with Solms’s (1997) observations about the role played by TPJ and mPFC in the sleep mentation production.

It should be underlined that these regions are involved in the “default mode network” (DMN), a neural system activated during resting state, daydreaming and mental imagery processes (Desseilles et al., 2011; Domhoff, 2011; Eichenlaub et al., 2014a). Mind wandering represents a state of attention toward interior feelings and personal thoughts, rather than processing items from the external environment (Smallwood and Schooler, 2006). This definition could be applied also to dreaming (Domhoff, 2011). A lot of studies evidenced that the mPFC, the anterior and posterior cingulate cortex, the precuneus and the temporo-parietal area are the regional substrate of mind wandering (Mason et al., 2007; Christoff et al., 2009). Specifically, Andrews-Hanna et al. (2010) observed that the DMN is organized in two subsystems: the dorsal mPFC system and the MTL system. In other words, mind wandering and dreaming both refers to a peculiar internal cognition, so it is plausible that the neural substrate for sleep mentation shares some brain regions with the network for mind wandering (i.e., DMN; Fosse and Domhoff, 2007; Ioannides et al., 2009; Nir and Tononi, 2010). Hence, it could be hypothesized that High Recallers are more interested in their inner word (Eichenlaub et al., 2014a). This aspect may represent a trait variable that can contribute to the episodic memory recall. Once again, the data appear consistent with the continuity hypothesis between mechanisms involved in cognitive processes across sleep and wakefulness. The same group demonstrated that High Recallers show a greater intrasleep wakefulness than Low Recallers (Ruby et al., 2013; Eichenlaub et al., 2014b). This result also provides some support to the arousal-retrieval model (Koulack and Goodenough, 1976). The model assumes that the information processing of dream mentation and the subsequent retrieval are facilitated if awakenings occur during sleep. The nocturnal awakenings could promote the transition of mnestic traces (i.e., dream contents) from short-term memory store to a long-memory storage (Koulack and Goodenough, 1976).

These studies have mainly investigated *interindividual* factors (structural features of amygdala and hippocampus). On the other hand, the *intraindividual* issue should be also considered, addressing the question of measuring DRF across different conditions. Some information on intraindividual differences comes from a recent study that performed a 40-h nap protocol under constant routine, awakening subjects every 75 min (Chellappa et al., 2009, 2011). This protocol ensures more possibilities to obtain recall or non-recall conditions in different sleep stages (NREM or REM sleep). However, this procedure raises other problems, concerning the influence of circadian and ultradian variables on dream activity. As matter of fact, it has been remarked that some components of dreaming vary as a function of chronobiological features (Nielsen, 2004, 2010). For instance, the length of dream reports shows a sinusoidal profile: NREM reports are longer when the awakening is closer to a prior REM sleep episode (Goodenough et al., 1965; Arkin et al., 1978). Furthermore, some features change in a circadian-dependent manner. Notable differences between dream reports from the first third and later parts of the night have been reported (Nielsen, 2004, 2010). In particular, sleep mentation increased in dream-like quality from the first REM to all later REM episodes (Kramer et al., 1980).

As mentioned above, despite some progresses in the dream research, the “state- or trait-like” question still remains unsolved. An alternative way to clarify this issue is represented by protocols with multiple sleep recordings in the same subject, carefully avoiding the influence of circadian factors. Our recent pilot study, applying a within-subject nap protocol, represents a first step toward this direction (Scarpelli et al., 2014). We have replicated the finding that dream recall subsequent the awakening from REM sleep is related to prior increasing of frontal theta oscillations (Marzano et al., 2011), providing further support to the “continuity hypothesis” between neurophysiological mechanisms for the encoding and retrieval of episodic memory in sleep and wakefulness (Marzano et al., 2011). More interestingly, these preliminary results suggest that dream recall, compared to absence of dream recall, is associated to a specific EEG pattern (frontal theta) during a specific sleep stage (REM sleep), supporting a “state-like hypothesis” (Scarpelli et al., 2014).

## Concluding Remarks

From the viewpoint of the neurosciences, dream research could be considered as the study of cognitive processes during sleep. However, neurobiological evidence is intrinsically limited by the impossibility to directly access to dreaming, and by the necessity to focus on recall of dream experience collected during post-awakening wakefulness (i.e., dream recall). A consistent number of studies underlined the continuity between mechanisms that regulate cognitive functions in wakefulness and sleep. In particular, here, it has been stressed that EEG studies have provided empirical data to sustain the predictive role played by theta oscillations in the retrieval of episodic information both in wakefulness (Klimesch et al., 1997, 2001; Klimesch, 1999; Kahana et al., 2001; Molle et al., 2002; Guderian and Düzel, 2005; Nyhus and Curran, 2010; White et al., 2013; Hsieh and Ranganath, 2014) and sleep, considering dream recall as an episodic mnestic trace (Marzano et al., 2011). Also neuropsychological studies reported a continuity across different states of consciousness. For instance, different types of dream activity alterations seem to have an equivalent in wakefulness (Solms, 1997). Neuroimaging investigations evidenced that amygdala and hippocampus are fundamental to consolidate and retrieve emotional memories in sleep as in wakefulness (De Gennaro et al., 2011). Furthermore, some structures of the DMN (i.e., mPFC and MTL) activated during internal cognition in wakefulness, are particularly activated also during sleep in subjects that recall more frequently dream contents (Domhoff, 2011; Eichenlaub et al., 2014a).

Taking into account the intrinsic difficulties in studying dream mentation, we have emphasized that PSG with laboratory awakenings should be still considered as a gold standard to investigate dreaming. The heterogeneous literature here discussed highlights that dream recall is associated to a particular EEG topography and frequency. We have also underlined that the existing literature does not provide compelling evidence to clarify the “state- or trait-like” factors influencing the neurobiological mechanisms of dream recall. Although some observations support the “state-like hypothesis,” only future studies could disentangle this issue by using designs with multiple within-subjects awakenings.

## Author Contributions

Substantial contributions to the conception and design of the work: LDG, MF, SS, ADA, MG; Drafting the work and revising it critically for important intellectual content: LDG, MF, SS, ADA, MG; Final approval of the version to be published: LDG, MF, SS, ADA, MG; Agreement to be accountable for all aspects of the work in ensuring that questions related to the accuracy or integrity of any part of the work are appropriately investigated and resolved: LDG, MF, SS, ADA, MG.

### Conflict of Interest Statement

The authors declare that the research was conducted in the absence of any commercial or financial relationships that could be construed as a potential conflict of interest.
